# A smartphone application for treating depressive symptoms: study protocol for a randomised controlled trial

**DOI:** 10.1186/s12888-018-1752-5

**Published:** 2018-06-01

**Authors:** M. Deady, D. A. Johnston, N. Glozier, D. Milne, I. Choi, A. Mackinnon, A. Mykletun, R. A. Calvo, A. Gayed, R. Bryant, H. Christensen, S. B. Harvey

**Affiliations:** 1 Black Dog Institute; Faculty of Medicine, UNSW, Sydney, Australia; 2 School of Psychiatry, UNSW Sydney, Sydney, Australia; 3 Central Clinical School, Brain and Mind Centre, Faculty of Medicine and Health, University of Sydney, Sydney, Australia; 4 School of Electrical and Information Engineering, University of Sydney, Sydney, NSW 2006 Australia; 5 School of Systems Management and Leadership, Faculty of Engineering and IT, University of Technology Sydney, Sydney, Australia; 6 Department of Mental Health and Suicide, Norwegian Institute of Public Health, Oslo, Norway; 7 School of Psychology, UNSW Sydney, Sydney, Australia; 8 MRC Cognition and Brain Sciences Unit, University of Cambridge, Cambridge, UK; 9 Department of Community Medicine, University of Tromsø, Tromsø, Norway; 10 Centre for Work and Mental Health, Nordland Hospital Trust, Bodø, Norway; 11 Centre for Research and Education in Forensic Psychiatry and Psychology, Haukeland University Hospital, Bergen, Norway

**Keywords:** Depression, Workplace, Mhealth, Treatment

## Abstract

**Background:**

Depression is a commonly occurring disorder linked to diminished role functioning and quality of life. The development of treatments that overcome barriers to accessing treatment remains an important area of clinical research as most people delay or do not receive treatment at an appropriate time. The workplace is an ideal setting to roll-out an intervention, particularly given the substantial psychological benefits associated with remaining in the workforce. Mobile health (mhealth) interventions utilising smartphone applications (apps) offer novel solutions to disseminating evidence based programs, however few apps have undergone rigorous testing. The present study aims to evaluate the effectiveness of a smartphone app designed to treat depressive symptoms in workers.

**Methods:**

The present study is a multicentre randomised controlled trial (RCT), comparing the effectiveness of the intervention to that of an attention control. The primary outcome measured will be reduced depressive symptoms at 3 months. Secondary outcomes such as wellbeing and work performance will also be measured. Employees from a range of industries will be recruited via a mixture of targeted social media advertising and Industry partners. Participants will be included if they present with likely current depression at baseline. Following baseline assessment (administered within the app), participants will be randomised to receive one of two versions of the *Headgear* application: 1) Intervention (a 30-day mental health intervention focusing on behavioural activation and mindfulness), or 2) attention control app (mood monitoring for 30 days). Participants will be blinded to their allocation. Analyses will be conducted within an intention to treat framework using mixed modelling.

**Discussion:**

The results of this trial will provide valuable information about the effectiveness of mhealth interventions in the treatment of depressive symptoms in a workplace context.

**Trial registration:**

The current trial is registered with the Australian and New Zealand Clinical Trials Registry (ACTRN12617000547347, Registration date: 19/04/2017).

## Background

Depression is now recognised as one of the leading causes of disability worldwide [1]. As well as being relatively common [2], depression can also be very debilitating [3], with core symptoms inclusive of; an absence of positive affect, persistent low mood, and low activity [4]. Persons with severe depressive symptoms report serious difficulties in all aspects of their life, including work, home, and social activities [5]. In response to the increased identification and recognition of depression [6], there has been greater emphasis on the development of innovative and effective psychological treatments.

The treatment of depression, both pharmacologically and with non-pharmacological interventions, remains a critical area of ongoing clinical research. There is substantial evidence supporting the efficacy of psychotherapy in the treatment of both sub-clinical [7] and clinical levels of depression [8]. Whilst cognitive behaviour therapy (CBT) remains the most empirically supported treatment [9], other therapies such as behavioural activation therapy (BAT) has been shown to be equally effective and also less complex to administer [10]. Service use statistics indicate that despite the effectiveness of such treatments, few people readily access these services [11]. Overcoming identified barriers to accessing psychotherapy such as cost, convenience and accessibility [12] may enhance people’s ability to access evidence based treatments.

Despite experiencing depression many people still remain as active participants in the workforce [13]. Ongoing employment is associated with numerous individual benefits such as economic, psychosocial, and emotional wellbeing [14]. Given its immediacy and core role in the lives of individuals, the workplace may be an avenue for providing psychological interventions to those who might not otherwise access mental health services in a conventional manner [15]. Interventions implemented in the workplace have been found to be effective in improving both mental health and occupational outcomes [16]. This highlights an added benefit of workplace interventions as medical interventions in isolation have not shown as positive an effect on work-related outcomes when compared to workplace interventions [17]. To date, most workplace interventions face accessibility and scalability issues, thus limiting the opportunity for employees to access these services. This issue appears to be particularly prevalent in workers employed in industries where roles are mobile and/or isolated; work hours are often intermittent or excessive; and tasks may be repetitive and high-risk (e.g. such as construction, transport and mining). These industries tend to be male-dominated (males > 70% of workforce) [18] and are associated with higher rates of mental health concerns [18]. Technological innovations, namely smartphones, provide an opportunity for individuals in such roles to learn how to manage their wellbeing and seek further support if required [19].

Mobile health (mhealth) technologies are increasingly being recognised as an effective means through which mental health interventions can be disseminated in the population [20, 21]. Such interventions overcome numerous barriers associated with treatment seeking, including stigma, cost, and accessibility [20]. Encouragingly, there is preliminary support that mhealth interventions can effectively treat symptoms in adults with depression [22]. Moreover, mhealth interventions offer the opportunity for an autonomous, user-directed approach that motivates and offers personalised support by allowing the delivery of content to be tailored to an individual’s interests and needs [23]. This may be particularly advantageous to workplace contexts where, individualised interventions are associated with more consistent outcomes than organisational wide interventions [24]. However, of the many mhealth interventions available on the market, few have been trialled [20] and even fewer have been specifically tailored to a workplace setting.

The proposed HeadGear trial aims to evaluate the effectiveness of a new smartphone app in treating depressive symptoms in a workplace context.

## Methods

### Design

The aim of the current study will be achieved through a multicentre randomized controlled trial, with two parallel arms. The trial will compare two smartphone app-based interventions: a novel intervention app designed for the treatment of depression (Headgear) and an attention control app (mood monitoring). Assessments will occur at baseline, post-intervention (5-weeks), and at 3-month follow-up. The study is registered with the Australian New Zealand Clinical Trials Registry (ACTRN12617000547347) and has ethical approval from the University of New South Wales (UNSW) Human Research Ethics Committee (HC17021). Consent to participate in the trial will be obtained electronically from all participants. The study will be conducted in accordance with the Helsinki Declaration [25] and is compliant with the CONSORT guidelines [26].

### Setting and participants

The study will recruit Australians who are currently employed, and will sample more selectively from a range of male-dominated industries [27]. In Australia, these industries include agriculture/forestry/fishing, utility services (electricity, gas, water and waste), wholesale trade, manufacturing, transport/postal/warehousing, mining, and construction [28]. Emergency services and defence also fit this definition, but were not considered unique industries by the ABS, though for this study they will also be considered as male-dominated.

Industry partner organisations will assist with recruitment by promoting the study among specific groups or their entire workforce. We will aim to recruit at least 850 employed adults across Australia. Organisations that elected to participate will promote the study via their respective health and wellbeing officers, along with email and newsletter advertisements. The study will also be promoted via members of the research team presenting at partner worksites. Social media advertising targeted at employed people will also be utilised to recruit individuals employed externally of partner organisations using an evidence-informed advertising approach [29]. Both males and females will be recruited.

### Eligibility criteria

Initial eligibility criteria are: having a valid telephone number, ownership of an Apple/Android-operating smartphone, fluent in English and living in Australia. As this trial is focused on the treatment of depression, participants will also be excluded from this trial (although still permitted to use the app) if they do not have substantial levels of depressive symptoms at baseline, as indicated by either a PHQ-9 score below 15, or the PHQ-9’s algorithm for a diagnosis for Major Depressive Disorder (MDD) not being met [30]. Participants were excluded if they were under 18 or not currently employed. No exclusion criteria were in place regarding comorbidities or medication use.

### Interventions

#### Active intervention: HeadGear

The intervention condition, *HeadGear*, is a smartphone application-based intervention utilising Behavioural Activation Therapy (BAT) and mindfulness-based therapies. Behavioural Action is a therapy based on learning theory that reconnects people to an environment of positive reinforcement, incorporating elements of value driven action and goal setting [31, 32]. When delivered face-to-face, BAT has been shown to perform as well as CBT for the treatment of depression [33] and preliminary evidence that this translates in mhealth form [34]. The other component utilised in Headgear, Mindfulness, draws on meditation practices to allow individuals to gain further insight into their emotional, physical, and/or cognitive experience to ultimately shape it [35]. Mindfulness has been identified as a transtherapeutic process that targets transdiagnostic mental processes [36], as evidenced by its effect in treating a range of psychopathologies including but not limited to mood, anxiety and substance use disorders [37, 38].

The therapeutic component of *HeadGear* encourages the user to complete one ‘challenge’ each day (5–10 min per day), over 30 days. These ‘challenges’ incorporate a variety of evidence-based BAT and mindfulness techniques and skills, including psychoeducational videos, value-driven activity planning and goal-setting, practice exercises, and techniques for developing coping and resilience (e.g., problem solving, improving sleep, minimising alcohol use, and/or assertiveness training). Other components of the Headgear intervention app include mood monitoring, a skill ‘toolbox’ (progressively built as the skills are completed), and a technical service helpline. Steps have been taken to promote user motivation and engagement by incorporating a ‘daily challenge’ framework where the user is ‘rewarded’ (through skill tokens) upon completing each daily challenge. A challenge framework has been shown to increase the general appeal of an app [39–41]. In addition to these elements, the Headgear application has been designed using participatory approaches and iterative human-computer interaction design strategies [42, 43].

#### Attention-matched control

The attention-matched control condition is a smartphone application that will have the same name and a virtually identical look and ‘feel’ as the intervention version of *Headgear and is* accessed in the same manner*.* However, there is no skill development and no component of behavioural activation or mindfulness therapy. To control for the attentional component of the *HeadGear* application, the control condition will encourage users to use the inbuilt mood monitor daily over a 30-day period and users will also have access to the ‘risk calculator.’

#### Procedure

**Fig. 1:**
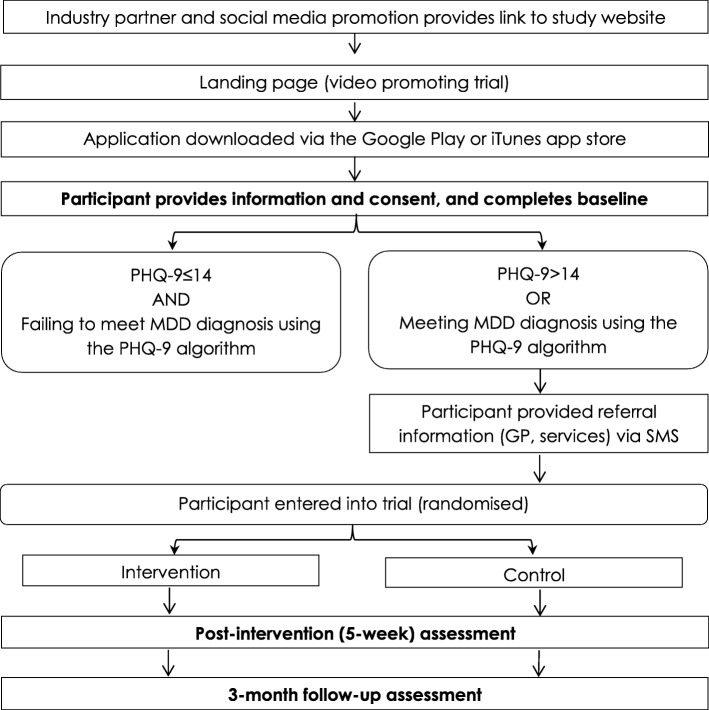
Flow of participants through the trial

All interested users will be directed to their respective app store (iTunes or Google Play) directly via a dedicated website (headgear.org.au) where participants register and provide their phone number. Informed consent will be sought digitally via both the website and the app itself. This provides information around the study aims, risks and benefits, confidentiality and dissemination of results. After consent and app download participants will undergo initial screening. Participants who meet the inclusion criteria will then be randomised to receive either the full *HeadGear* app or the attention-matched control version of the app. Participants will be blinded to their allocation. All participants will be provided with appropriate referral information to health services and crisis lines and will be encouraged to seek help from their GP (if this has not already occurred) while completing the trial. The flow of participants through the study phases is shown in Fig.1.

### Random allocation

Randomisation will occur immediately following completion of the baseline assessment using automated procedures integrated into the trial management software. The algorithm for randomisation will consist of a block design, stratified by industry type, with a block size of 10.

### Assessment

#### Administration of assessments

Assessments will be completed at baseline, post-intervention (5-weeks), and 3-month follow-up. Baseline assessment includes outcome measures pertaining to depression symptomatology (Patient Health Questionnaire (PHQ-9) [44]), wellbeing (World Health Organisation-5 (WHO-5) Well-Being Index [45, 46]), anxiety symptomatology (General Anxiety Disorder-2 item (GAD-2) [47], resilience (Connor-Davidson Resilience Scale 10-item (CD-RISC10) [48]), work performance and absenteeism (Health and Work Performance Questionnaire (HPQ) [49]). In addition to these measures, demographic and service use information will be collected. The application monitors usage data including number of log-ins, frequency of use, time spent in-app, and activity completion rates. This data will be used to examine program engagement.

Post-intervention assessment will occur at 5 weeks’ post-baseline, to allow users extra time to complete the 30-day program. Participants will be reminded to complete the post-intervention assessment via text messages to phone numbers provided in order to address for the possibility that users may delete the app during the trial. Participants will complete an online questionnaire similar to the baseline measure (see Table 1), and again at 3 months post-baseline (“3-month follow-up”). Participants will receive up to three text messages and a call at each follow-up assessment time point, linking them to an online platform through which they can complete the assessment. On completion of each assessment, participants will be entered into a draw to win one of four $200 Visa gift cards.

**Table 1:** Assessment measures

	Baseline	Post-intervention	3-month follow-up
Demographics	×		
Patient Health Questionnaire-9 item (PHQ-9) [44]	×	×	×
General Anxiety Disorder-2 item (GAD-2) [47]	×	×	×
World Health Organisation-5 (WHO-5) Well-Being Index [45, 46]	×	×	×
Connor-Davidson Resilience Scale 10-item (CD-RISC10) [48]	×	×	×
Health and Work Performance Questionnaire (HPQ) [49]	×	×	×
Service utilisation and management items	×	×	×
Program feedback		×	

#### Specific measures used in online assessments

The PHQ-9 will be used to measure depression symptomatology [50]. The PHQ-9 is a reliable and valid nine-item measure of depression severity over the past 2 weeks [44, 51]. Each of the nine items of the PHQ-9 is scored as 0 (not at all), 1 (several days), 2 (more than half the days), or 3 (nearly every day). Scores are summed to provide a total score (score range 0–27 with 0 indicating no depressive symptoms and 27 indicating all symptoms occurring nearly daily). The criterion and construct validity of the PHQ-9 has previously been demonstrated, with 73% sensitivity and 98% specificity in detecting major depression compared to clinician-based assessment [28, 50] and, regardless of diagnostic status, typically represents clinically significant depression [50]. The measure has excellent internal consistency (Cronbach *α* > 0.85 in multiple samples) and test-retest reliability (α = 0.84) [52].

Anxiety will be measured using the 2-item Generalised Anxiety Disorder scale (GAD-2) [47]. The GAD-2 consists of the two core criteria for generalized anxiety disorder, which have been shown to be effective screening items for panic, social anxiety, and post-traumatic stress disorders [47]. The GAD-2 begins with the stem question: “Over the last 2 weeks, how often have you been bothered by the following problems?” Response options are “not at all”, “several days”, “more than half the days”, and “nearly every day”, scored as 0, 1, 2, and 3, respectively (total ranging from 0 to 6). A total scale score ≥ 3 is suggested as a cut-off point between the normal range and “probable anxiety” [47].

Resilience will be measured by the 10 item Connor-Davidson Resilience Scale (CD-RISC-10) [53]. The CD-RISC-10 is a self-rated measure, with each question rated on a 5-point scale from 0 (‘not true at all’) to 4 (‘true nearly all the time’). The CD-RISC-10 has been shown to differentiate between individuals who function well after adversity and those who do not and measures the core features of resilience and the ability to tolerate experiences [53]. It is believed that increased resilience may reduce rates of mental ill health [54].The scale demonstrates high internal consistency (Cronbach’s α = 0.89), construct validity, and test-retest reliability in the general population and in clinical settings [48]. Total scores range from 0 to 40 with higher scores corresponding to greater resilience. The scale has been shown to have good concurrent validity, with higher resilience on the scale associated with lower levels of perceived stress [48] Validity is high relative to other measures and reflects differentiation in resilience among diverse populations, showing that higher levels of resilience are consistent with lower levels of perceived stress vulnerability [48].

Wellbeing will be assessed using the 5-item WHO Wellbeing Index (WHO-5) [45, 46]. Participants are asked to self-report on the presence or absence of wellbeing on a 6-point scale ranging from 5 (‘all of the time’) to 0 (‘none of the time.’) Raw scores range from 0 to 25 where 0 indicates the worst possible quality of life while a score of 25 represents the best possible quality of life. A score ≤ 13 or an answer of 0 or 1 on any of the five items shows poor wellbeing. WHO-5 is a psychometrically sound measure of well-being with high internal consistency (Cronbach’s α = 0.84) and convergent associations with other measures of well-being [55].

Work Performance will be measured using three items (performance items A10, A11, A12) from the Health and Work Performance Questionnaire (HPQ) [49]) and two additional items pertaining to: i) sickness absence over the past month (days absent more generally, and days absent specifically for mental health reasons), and ii) weeklong sickness absence over the past 6-months (weeks absent more generally, and weeks absent specifically for mental health reasons).

Service use and management items comprised of seven items assessing lifetime and past month service use, along with current medication use. Participants were also asked about their abilities (perceived capability and effectiveness) to manage their mental fitness, and autonomy (choice and freedom) in management. These were scored on a 7-point Likert scale from strongly disagree to strongly agree.

#### Safety protocol

In any trial concerned with mental health, there is the potential for participants to experience psychological distress. Those who meet criteria for the trial will within the app trigger the user to be directed to a “get support” page (at each assessment point) and will suggest the participant seek further help from these support services or their general practitioner (GP). Additionally, an optional call-back service for individuals requiring further support or direction is provided. This call-back will be conducted by a team-member with psychology training, within 4 days, with the purpose to guide participants into necessary care arrangements. If the team member still has concerns for the participant’s safety, an accredited psychiatrist will contact the participants within the next 24 working hours. Participants will also receive an SMS with a range of support service contacts, and another reminder to consult with their GP regarding their mental health.

#### Study hypotheses and outcomes

We hypothesise that participants receiving the *HeadGear* intervention will have reduced levels of depression symptomatology at post-intervention and 3-month follow-up, compared to participants in the attention-matched control condition. While the primary analyses will be conducted on the entire sample (to examine the intervention effect). We also predict the intervention effect to vary according to the level of depression symptoms at baseline [56], with a greater effect amongst those with higher levels of symptomatology.

Secondly, it is hypothesized that—relative to the control group—*HeadGear* Intervention participants will have lower rates of depressive disorder as detected by the PHQ algorithm at all follow-up time points. We also hypothesise that the intervention group will have reduced levels of anxiety symptomatology, and improved wellbeing, resilience, and work performance, at all follow-up time points, relative to controls.

#### Primary outcome

The primary outcome measure of the study will be the level of depressive symptomatology (as measured by the PHQ-9) at the 3-month follow up period.

#### Secondary outcomes

A range of secondary outcomes will be considered, including change in anxiety symptomatology (as measured by the PHQ-2) at both 5-week and 3-month follow up. Other secondary outcomes include incident caseness of depression at 5-week and 3-month follow-up (as measured by the PHQ-9 diagnostic algorithm), and the level of depression symptoms (PHQ-9) at 5-week follow up. Finally, change in Wellbeing (as measured by the WHO-5) and change in occupational functioning (as measured by the HPQ and sickness absence questions) at both 5-week and 3-month follow-ups will be outcomes of interest.

### Statistical analysis

#### Analysis plan

Primary analyses will be undertaken on an intent-to-treat basis, including all participants as randomised, regardless of treatment received or withdrawal from the study. Likelihood based methods (mixed-model repeated measures (MMRM)) methods will be favoured to analyse change in the primary outcome measure (PHQ-9). A priori planned comparisons of change from baseline across the 3-month follow up period will be used to test the primary hypothesis. An unstructured variance-covariance matrix will be used to accommodate relationships between observations at different occasions. Variables found to be substantially imbalanced between groups post randomisation will be tentatively included in these models and retained if statistically significant and influential on outcomes. Similar analyses of scaled secondary measures will assess differential change due to intervention arm. Mathematical transformation or categorisation of raw scores may be undertaken to meet distributional assumptions and address any violation of assumptions attributable to outliers.

Baseline characteristics will be used to define subgroups that would be the targeted if the app were offered as treatment. Group membership will be used for models to evaluate moderation of effectiveness by adding appropriate interaction terms and undertaking planned comparisons. The effect on outcome of level of baseline depressive symptom levels, level of functional improvement and recruitment method at baseline will be explored using an analysis of covariance approach using baseline measures as a covariate and including a covariate by intervention arm interaction term in models. The effectiveness of the active intervention at clinically relevant levels of baseline covariates will be assessed using planned comparisons while the lowest values of covariates associated with a significant benefit of the intervention will be established using a Johnson and Neyman [57] approach.

All tests of treatment effects will be conducted using a two-sided alpha level of 0.05 and 95% confidence intervals.

#### Sample size

As a treatment, the size of the effect of the intervention is anticipated to be moderate. Meta-analysis of previous trials of internet and mobile based treatment of depression showed a large effect size of g = − 0.90 [22]; however unguided interventions typically show smaller effect sizes. Power calculations were carried out using the R package simR [58]. Power was set at 80%, alpha at 0.05, 2-tailed tests, and a correlation of .50 between pre- and post- intervention scores was assumed. Based on these calculations, a sample size of 266 per group was needed (total *N* = 532). A conservative dropout rate of 40% at follow-up was estimated. An initial sample of 851 will therefore be recruited for the trial.

#### Dissemination

Results of this study will be disseminated for publication in peer-reviewed journals and key findings presented at national and international conferences.

## Discussion

This study is planned to be the largest randomised controlled trial of a smartphone intervention for depression. By targeting the workers, this trial will provide valuable evidence regarding the effectiveness of *m*health interventions in treating depressive symptoms in a workplace context. Given the substantial impact that depression has on the individual and the employer, if shown to be effective, this program would allow for a simple and economical means by which an organisations or governments could disseminate a tailored intervention for workers. Given the proliferation of untested smartphone applications, the dissemination of evidence based products into the workplace, and indeed the wider community, remains a pressing need.

Employees with significant depressive symptoms have higher rates of absenteeism, presenteeism and job turnover [59]. Remaining in the workforce is important as it offers structure, empowerment, financial security and protection from the psychological impacts of unemployment [14]. Using the workplace as a means to dispense or promote an intervention may not only be protective for the individual concerned, but may also assist in overcoming the issue of individuals delaying or not receiving treatment [60]. From an economic standpoint, it is has been established that, for the workplace, such an approach is more cost-effective for the organisation [61]: utilising smartphone technologies would improve upon this cost-effectiveness. It is also hoped that dissemination via workplaces and social media will help engage individuals who would not usually access help via the health care system.

This study will provide valuable evidence regarding the effectiveness of mhealth tools in the treatment of depressive symptoms. Despite the proliferation of mental health apps, there is scarce research on the effectiveness of such apps. Indeed, in a systematic review of the literature on smartphone interventions, only five mental health apps were empirically tested and only one of these did not require the input from a mental health professional [20]. mHealth interventions offer advantages in that they increase user autonomy and anonymity, which may be important as stigma [62] and lack of knowledge of services [63] can impact upon help-seeking behaviour in the workplace. An intervention developed for the workplace also carries the benefit in that it could ameliorate some of the financial burden placed solely on the healthcare system.

The proposed trial does carry with it a number of limitations. The use of a smartphone app as a delivery modality does mean that the intervention is unguided and that the user is responsible for managing their interaction with the program. Thus, trial attrition and disengagement are expected issues [64, 65]. It is worth noting, however, that this has also been an issue for face-to-face psychotherapy trials [66]. The reason for drop out in both mhealth and face-to-face trials is often multi-faceted, and whilst can be related to engagement with the program, it is rarely only due to dissatisfaction [67].To account for potential drop out, two procedures were put into place. Firstly, all follow-up communication would occur via phone numbers to ensure that if the participant uninstalled the app before the follow-up period, the participant could still be reached. Secondly, conservative drop-out estimation and the use of statistical methods robust to data missing at random, it is believed that this limitation will be minimised.

Another limitation of the present trial is related to the reliance on self-reported depressive symptoms, rather than a diagnosis of depression achieved through a structured diagnostic interview. This is a common issue faced by most similar trials given the constraints around time and resources. To overcome this issue, we will use a well-validated measure (PHQ-9) that contains two methods for classifying depression: 1) threshold total score above 14 (sensitivity = 67%; specificity = 95%) [68] or 2) meeting the depression algorithm’s criteria (sensitivity = 0.53; specificity = 0.94) [69]. By using both methods, we are confident that participants without significant symptomatology will be excluded and those with significant symptomatology will be included.

The treatment of depression utilising evidence based *m*health interventions remains an important area of clinical research. The Headgear trial will be the largest trial of a smartphone application that seeks to offer an alternate or augmentation to traditional face-to-face therapy through which working adults can manage their mental health and wellbeing. This trial will be unique in that it is advertising a treatment via a workplace setting, and allowing for the assessment of clinical and occupational outcomes. Finally, the Headgear Trial will provide much needed information on the general effectiveness of evidence-based interventions (BAT and mindfulness) delivered through smartphone technology.
